# Metabolic Fingerprinting for Identifying the Mode of Action of the Fungicide SYP-14288 on *Rhizoctonia solani*

**DOI:** 10.3389/fmicb.2020.574039

**Published:** 2020-12-09

**Authors:** Li Liang, Xingkai Cheng, Tan Dai, Zhiwen Wang, Jin Li, Xueming Li, Bin Lei, Pengfei Liu, Jianjun Hao, Xili Liu

**Affiliations:** ^1^Department of Plant Pathology, China Agricultural University, Beijing, China; ^2^Institute of Nuclear and Biological Technologies, Xinjiang Academy of Agricultural Sciences, Urumqi, China; ^3^School of Food and Agriculture, University of Maine, Orono, ME, United States

**Keywords:** uncoupler, ATP synthase inhibitor, membrane potential of mitochondria, respiration inhibitor, phosphorylation inhibitor

## Abstract

The fungicide SYP-14288 has a high efficiency, low toxicity, and broad spectrum in inhibiting both fungi and oomycetes, but its mode of action (MoA) remains unclear on inhibiting fungi. In this study, the MoA was determined by analyzing the metabolism and respiratory activities of *Rhizoctonia solani* treated by SYP-14288. Wild-type strains and SYP-14288-resistant mutants of *R. solani* were incubated on potato dextrose agar amended with either SYP-14288 or one of select fungicides acting on fungal respiration, including complex I, II, and III inhibitors; uncouplers; and ATP synthase inhibitors. Mycelial growth was measured under fungicides treatments. ATP content was determined using an ATP assay kit, membrane potential of mitochondria was detected with the JC-1 kit, and respiratory rate was calculated based on the measurement of oxygen consumption of *R. solani.* A model of metabolic fingerprinting cluster was established to separate oxidation inhibitors and phosphorylation inhibitors. All the results together displayed a clear discrimination between oxidation inhibitors and phosphorylation inhibitors, and the latter inhibited ATP synthase production having or uncoupling activities. Based on the model, SYP-14288 was placed in phosphorylation inhibitor group, because it significantly reduced ATP content and membrane potential of mitochondria while increasing respiratory rate in *R. solani*. Therefore, the MoA of SYP-14288 on *R*. *solani* was confirmed to involve phosphorylation inhibition and possibly uncoupling activity.

## Introduction

The soil-borne fungus *Rhizoctonia solani* is a ubiquitous plant pathogen that attacks almost all known crops, pastures, and horticultural species, leading to significant yield losses (El-Samawaty et al., 2012; Constable and Bange, 2015; Anderson et al., 2016; Ajayi-Oyetunde and Bradley, 2018). Many fungicides have been applied in controlling *R. solani*. The fungicide SYP-14288, or 2,4-dinitro-5-chloro-[1-(2,6-dichloro-4-nitroaniline)]-toluene (Sinochem Agrochemicals R&D Co., Ltd., Shenyang, China), exhibits a high level of fungitoxicity against *R. solani* and many other pathogens (Lan et al., 2012; Cai et al., 2019). Since it has been internationally patented, we anticipate that it will have great potential use and large market share for field disease control (Zhang and Liang, 2017).

SYP-14288 belongs to the diarylamine group and shares a similar chemical structure with fluazinam, which possibly interrupts cellular energy production by uncoupling mitochondrial oxidative phosphorylation (Wang et al., 2018; Hou et al., 2019). Compared with fluazinam, SYP-14288 possesses a higher efficacy and has broader spectra for disease control. Because fluazinam and SYP-14288 are structurally similar, one would consider that they have the same mode of action (MoA). However, there are no data to support this hypothesis. A previous study had shown that SYP-14288 influenced the respiratory process and inhibited ATP biosynthesis in *Phytophthora capsici* (Wang et al., 2018), but further investigation is still needed, especially on different pathogens, such as *R. solani*. This is because *P. capsici* belongs to oomycetes, but *R. solani* is a member of true fungi. Therefore, the MoA of SYP-14288 on *R. solani* needs to be confirmed.

Mode of action is a key for understanding the mechanism of fungicides in inhibiting pathogens (Jin et al., 2013). Understanding the MoA of fungicides helps in effectively managing fungicide resistance and increasing efficacy of disease control (Gisi and Sierotzki, 2008). Exploring the MoA of fungicides can be done by using classical biochemical methods (Mitani et al., 2001), such as fungitoxicity assay combined with DNA sequence analysis of the target gene and molecular docking of ligands at the quinone inside site used for studying fenpicoxamid on *Zymoseptoria tritici* (Young et al., 2018).

More analytical techniques have been applied for the toxicological evaluation on novel chemicals and especially discovery of targeting sites of fungicides (Mei et al., 2014). For example, isobaric tags, a proteomics approach, has been used in measuring relative and absolute quantitation (iTRAQ) of pyrimorph (Pang et al., 2015). The combination of immunofluorescence analysis, classical biochemistry, and bioinformatics has offered the possibility of generating a first insight for describing the MoA of a novel fungicide fluopicolide (Toquin et al., 2009) to accelerate the identification of the biochemical MoA of novel fungicides.

Metabolomics is a fast-growing and high-throughput platform for bioanalysis applied in studying MoAs of many microbes such as yeast, *Saccharomyces cerevisiae* (Allen et al., 2004), and pathogenic fungi (Aliferis and Jabaji, 2011; Sevastos et al., 2018). This method has unique advantages over genomics, transcriptomics, and proteomics because the metabolome indicates what is actually happening in a biological system, thus serving as a link between genome and phenome (Fiehn, 2002; Wiklund et al., 2008; Carreno-Quintero et al., 2013). Metabolomics encompasses qualitative and quantitative characterizations of target organisms (Cai et al., 2015).

Gas chromatography–mass spectrometry (GC-MS) has been used to elucidate the MoAs of fungicides (Hu et al., 2019). In this method, when the pathogen (*Botrytis cinerea*) was treated by fungicides, induced metabolic perturbations can be observed and used to establish a comprehensive database of metabolic fingerprinting. Different metabolic patterns resulted from fungicides with different MoAs. Therefore, the MoA of different fungicides can be identified based on the database. Although it can only discriminate MoAs among known groups documented by the Fungicide Resistance Action Committee (FRAC, http://www.frac.info/) and cannot easily determine MoAs in subgroups such as groups under respiration inhibitors, the technique has a great potential in the study of MoA of fungicides. The objectives of this study were to establish a method using metabolic fingerprinting and use it for fast identification of MoAs of SYP-14288 affecting *R. solani*.

## Materials and Methods

### Fungal Isolates and Chemicals

*Rhizoctonia solani* strain X19 was collected from rice plant and confirmed to be AG1-IA using anastomosis group (AG) analysis. SYP-14288-resistant mutants X19-2 and X19-7, with resistance factors being 125.7 and 56.7, were obtained through domestication of the parental strain X19 by exposing to SYP-14288, which was reported previously (Cheng et al., 2020). Briefly, mycelial plugs were incubated on potato dextrose agar (PDA) plates containing SYP-14288 at 0.05 mg/ml. Newly grown mycelial colonies were selected and transferred to a new PDA plate containing SYP-14288 at 0.05 mg/ml for mutation confirmation. All fungicides used in this study were of pure technical grades (Table 1). Fungicides were dissolved in dimethyl sulfoxide (DMSO) to make stock solutions (1 × 10^5^ μg/ml) and stored at 4°C until use. Analytical grade of *N*,*O*-bis(trimethylsilyl)trifluoroacetamide (contains 1% trimethylchlorosilane, 99%), pyridine (99%), methoxyamine hydrochloride (99%), and salicin (99%) were purchased from Sigma-Aldrich (Steinheim, Germany). Methanol (chromatographically pure) was obtained from Sinopharm Chemical Reagent Co., Ltd. Ultrapure water was prepared using the Milli-Q Academic A10 water purification system from Millipore (Billerica, MA, United States).

**TABLE 1:** Fungicides used in the study.

Fungicide	Mode of action	Active ingredient (%)	Distributor	Working concentration (μg/mL)*
Diflumetorim	Complex I inhibitor	90.0	Shenyang Research Institute of Chemical Industry Co., Ltd.	10.0
Carboxin	Succinate DeHydrogenase Inhibitor (SDHI)	97.0	Jiangsu Huifeng Agrochemical Co., Ltd.	0.05
Thifluzamide	Succinate DeHydrogenase Inhibitor	96.0	Zhejiang Heben Pesticide & Chemicals Co., Ltd.	0.05
Azoxystrobin	Quinone outside inhibitor	98.0	Jiangyin Suli Chemical Co., Ltd.	0.5
Pyraclostrobin	Quinone outside inhibitor	95.0	Shandong United Pesticide Industry Co., Ltd.	0.05
Cyazofamid	Quinone inside Inhibitor	95.0	Beijing Mindleader Agroscience Co., Ltd.	10.0
Fentin chloride	ATP Synthase Inhibitor	95.0	Heowns Biochem Technologies LLC	5.0
Fentin acetate	ATP Synthase Inhibitor	98.0	Hubei Kangbaotai Fine Chemical Co., Ltd.	5.0
Oligomycin A	ATP Synthase Inhibitor	98.0	Kamai Shu Biotechnology Co., Ltd.	1.0
2,4-dinitrophen	Uncoupler	99.9	Heowns Biochem Technologies LLC	12.0
Mefenamic acid	Uncoupler	98.0	Heowns Biochem Technologies LLC	10.0
Fluazinam	Uncoupler	98.0	Beijing Mindleader Agroscience Co., Ltd.	0.25
Difenoconazole	DeMethylation Inhibitor	95.0	Ningbo Sunjoy Cropscience Co., Ltd.	–
SYP-14288	Unknown	98.0	Shenyang Research Institute of Chemical Industry Co., Ltd.	0.05

*The concentration of fungicides used for the establishment of metabolic fingerprinting cluster model, and “–” means not used in the model.

### Metabolomic Analysis of Fungal Mycelia

#### Preparation of Fungicide-Treated Mycelia

Typical respiration inhibitors (fungicides) having five different MoAs were used for establishing metabolic models. Cultural plugs (5 mm in diameter) cut from the periphery of a 3-day-old colony of X19 were placed on PDA plates amended with the above respiration inhibitors to obtain a series of final concentrations. For azoxystrobin and pyraclostrobin, 100 μg/ml of salicylhydroxamic acid (SHAM) was added to suppress the alternative oxidase pathway. The concentration of DMSO in the medium was adjusted to 0.1%. Plates amended with only DMSO at the final concentration of 0.1% in volume were used as a control. The plates were incubated at 25°C in the dark for 2 days, and radial mycelial growth was measured by measuring colony diameter perpendicularly. Mean colony diameter (minus the diameter of the inoculation plug or 5 mm) was measured and expressed as a percentage of growth inhibition (the percentage of growth inhibition determined relative to the fungicide-free check; Mu et al., 2017). Concentrations of different fungicides for 40% to 60% mycelial inhibition were selected, and mycelial plug from the periphery of 3-day-old colony was placed on the center of PDA plates amended with the fungicides. A piece of sterilized glass paper was used to cover the medium and separate from the cultural plug. The culture was incubated for 3 days at 25°C in darkness. Mycelia were harvested and transferred to 2-ml centrifuge tubes, frozen with liquid nitrogen, and stored at −80°C until use. Each concentration was replicated six times. The procedure was performed twice.

#### Chemical Analysis

Mycelial metabolomes were extracted from *R*. *solani* X19, which was sensitive to all test fungicides. Metabolome extraction and derivatization were based on previously described methods with slight modifications (Hu et al., 2019). Mycelia prepared as described above were taken out from −80°C freezer and freeze-dried by Christ freezer (Alpha 1-2 LD plus, Osterode, Germany), and homogenized powder was obtained by shaking in a ball mill (Retsch, Haan, Germany) at 30 times per seconds for 2 min. Then, 30-mg powder was placed into 2-ml centrifuge tubes and suspended with 1.8 ml of extraction solution [salicin was added to methanol/water (80/20, v/v) to obtain a final concentration of 10 μg/ml]. The sample was vortexed vigorously by MX-F vortex mixer (Dragon Laboratory Instruments Co. Ltd., Beijing, China) for 1 min and then placed in an ultrasonicate (Kunshan Ultrasonic Instruments Co. Ltd., Kunshan, China) for 20 min. Finally, samples were centrifuged by ultracentrifuge (Thermo Scientific, Wilmington, United States) at 12,000 *g* for 15 min. An aliquote of 0.6 ml of supernatant containing fungal metabolites was dried in a vacuum centrifuge (Hunan Herexi Instrument & Equipment Co. Ltd., Hunan, China) at 45°C and maintained for further analyses. The metabolome extraction step was conducted six times.

For derivatization, 100 μl of methoxyamine hydrochloride dissolved in pyridine at 20 mg/ml was added to the dried extracts followed by incubation at 30°C for 2 h. Then, 100 μl of *N*,*O*-bis(trimethylsilyl)trifluoroacetamide was added, and the mixtures were incubated at 37°C for 6 h. After centrifugation at 12,000 *g* for 15 min, 160 μl of supernatant was transferred to GC-MS autosampler vial for detection. All of the samples were detected within 48 h. The GC-MS analysis was performed on a QP 2010 GC (Agilent Technologies 7890 B)–MS (Agilent Technologies 7693) system equipped with a HP-5MS capillary column (30 m × 0.32 mm × 0.25 μm). The column temperature was initially adjusted to 60°C for 2 min, increased to 325°C at a ratio of 10°C/min, and held for 10 min. The temperature of ion source and interface was set to 250°C and 290°C, respectively. Helium was used as carrier gas with a constant flow at 1 ml/min. Electron ionization source was 70 eV. The MS scan parameters were set as follows: a mass scan range of m/z from 50 to 600, a scan interval of 0.2 s, and a detector voltage of 0.9 kV. Sample (1 μl) was injected by the Agilent autoinjector, and the acceleration voltage did not work until a solvent delay for 5.9 min.

On the chromatograph, peak area was calculated for each sample. The difference of peak areas between a fungicide treatment and non-treated control was logarithmically transformed for hierarchical cluster analysis (HCA), using Ward’s linkage (clustering to minimize the sum of squares of any two clusters) and Euclidean distance (Ward et al., 2010; Mumm et al., 2016). A *t*-test was performed between fungicide-treated and non-treated samples. A higher peak area of metabolites for a fungicide treatment indicated an up-regulated metabolite, and a lower peak area indicated a down-regulated metabolite. A metabolite was determined and selected as a biomarker that discriminated between oxidation and phosphorylation inhibitors under one of the two following conditions: (1) the metabolite was all either up- or all down-regulated under all oxidation inhibitors but not phosphorylation inhibitors. (2) The metabolite was all either up- or all down-regulated under all phosphorylation inhibitors but not oxidation inhibitors.

### Effect of SYP-14288 on ATP Production of *Rhizoctonia solani* Mycelia

The wild-type isolate X19 and its derived SYP-14288-resistant mutants X19-2 and X19-7 were cultured in potato dextrose broth (PDB) at 25°C for 24 h, followed by adding one of the test fungicides at final concentrations of 0.01 and 0.1 μg/ml for SYP-14288, 10 and 25 μg/ml for 2,4-dinitrophen, 0.25 and 1.00 μg/ml for fluazinam, 0.10 and 0.25 μg/ml for fentin chloride, and 0.05 and 0.25 μg/ml for azoxystrobin. Difenoconazole, which does not affect ATP production, was used as negative control with the final concentrations of 0.05 or 0.10 μg/ml. DMSO was used as a blank control. Mycelia were continuously cultured for another 24 h and then collected and freeze-dried to obtain homogenized powder. The powder (30 mg) was suspended in 600 μl of enzyme solution (40 mg/ml lysing, 30 mg/ml cellulose, and 40 mg/ml of snailase were dissolved in STC isosmotic solution) in a 2-ml centrifuge tube. The tube was centrifuged at 12,000 *g* for 5 min at 4°C. About 450 μl of supernatant was added to 1.5-ml centrifuge tubes for later use. ATP content in mycelium was measured using ATP assay kit by following the manufacturer’s instructions (Beyotime, Shanghai, China). ATP concentration was calculated in a form of nmol/g protein, and the protein concentration was determined based on the bicinchoninic acid assay (BCA) method (CWBIO, Beijing, China). Each treatment was represented by three replicates, and the experiment was performed twice.

### Effect of SYP-14288 on *Rhizoctonia solani* Respiratory

Wild-type isolate X19 and SYP-14288-resistant mutants X19-2 and X19-7 of *R. solani* were grown on PDA plates covered with glass paper. Mycelia (0.03 *g*) were added to an oxygen electrode reaction cup containing 2 ml of 2% glucose solution, followed by the addition of 2 μl of SYP-14288, which was adjusted to final concentrations of 0.005, 0.01, 0.1, or 0.5 μg/ml. As positive controls, fentin chloride, 2,4-dinitrophen, and fluazinam were added to the cups to attain final concentrations of 0.1, 0.5, 1, or 5 μg/ml; 1, 5, 10, or 25 μg/ml; and 0.01, 0.1, 1, or 2.5 μg/ml, respectively. Azoxystrobin and difenoconazole were used as negative controls, with final concentrations of 0, 0.1, 0.5, 5, or 10 μg/ml; and 0, 0.05, 0.5, 5, or 10 μg/ml, respectively. Control cups were treated with 2 μl of DMSO. Oxygen consumption and the respiratory rate were recorded by the change in the rate of respiration (PR), which was calculated using the following formula: PR = (R_1_ − R_0_)/R_0_, where R_1_ is the respiratory rate of treatment and R_0_ is the respiratory rate of control. Each treatment was represented by three replicate cups, and the experiment was performed twice.

### Effect of SYP-14288 on Mitochondrial Membrane Potential of *Rhizoctonia solani*

Mitochondrial membrane potential (MMP) of *R. solani* protoplasts was detected with the JC-1 kit (Beyotime Institute of Biotechnology, Beijing, China). To prepare protoplasts, wild-type isolate X19 and SYP-14288-resistant mutant X19-7 were cultured in NPB (1 *g* of K_2_HPO_4_, 3 *g* of KNO_3_, 2 *g* of CaCO_3_, 0.1 *g* of CaCl_2_, 0.5 *g* of MgSO_4_⋅7H_2_O, 5 *g* of D-mannitol, 5 *g* of D-sorbitol, 5 g of glucose, 2 *g* of yeast extract, 2 ml of vitamin stock, and 2 ml of trace elements in 1 L of distilled pea broth). The mycelium was collected and rinsed three times with 0.8 mol/L of mannitol followed by filtration with gauze and treated with compound enzyme hydrolysate for 6 h at 37°C. The obtained protoplasts were filtered by two layers of Miracloth and washed with STC isosmotic solution. Protoplasts were collected in 50-ml centrifuge tubes and suspended in STC isosmotic solution. SYP-14288 (1 μl) was diluted with 500 μl of protoplast suspension to attain final concentrations of 0.01, 0.02, 0.1, or 0.2 μg/ml for 1.5 h at 25°C in darkness, followed by 500 μl JC-1 dyeing solution for another 0.5-h incubation. Fluazinam, fentin chloride, 2,4-dinitrophen, azoxystrobin, and difenoconazole with final concentrations of 0.2, 1, 2, or 5 μg/ml; 0.2, 0.5, 2, or 5 μg/ml; 10, 20, 50, or 100 μg/ml; 0.2, 1, 2, or 5 μg/ml; and 0.2, 1, 2, or 5 μg/ml, respectively, were used as control fungicides. Protoplasts were precipitated and rinsed using JC-1 staining buffer, and 200 μl of samples was added for the fluorescence intensity by a multifunctional microplate reader (i3x, Molecular Devices) after resuspension with an appropriate amount of JC-1 staining buffer. The ratio of red and green fluorescence intensity was used to characterize the change of MMP with the treatment of fungicides. DMSO was used as blank control, while uncoupler carbonyl cyanide *m*-chlorophenylhydrazone (CCCP), which could cause the loss of MMP, was regarded as the positive control. Each treatment was set up in triplicate, and the experiment was performed twice.

### Statistical Analyses

#### Fungal Growth Data

Data analysis was performed using SPSS software (SPSS V22, IBM SPSS Statistics, Chicago, IL, United States). Prior to analysis, data expressed as percentages were arcsine transformed to homogenize variances. The effects of different treatments were examined using analysis of variance (ANOVA), and when the *F*-test was significant at *P* ≤ 0.05, treatment means were compared using Fisher’s least significant difference (LSD).

### Gas Chromatography–Mass Spectrometry Data

Gas chromatography–mass spectrometry data were acquired by using the software Qualitative Analysis B.07.00 (Agilent Technologies, Santa Clara, CA, United States) and following procedures established in our previous research (Fiehn et al., 2007 and Hu et al., 2019). All detected metabolites were identified by comparing spectra with the NIST14 library and Fiehn database (Kanani et al., 2008). Instrumental stability was evaluated by analyzing the percentage of relative standard deviation (RSD) of the internal standard substance (>99% salicin, Sigma-Aldrich) in the six repeated tests. The SPSS Statistics 21 software (IBM, Armonk, United States) was used to analyze metabolites with one-way ANOVA followed by a Tukey test at a significance level of *P* < 0.05. HCA was carried out based on average areas calculated from principal ions of all the compounds.

## Results

### Metabolic Fingerprinting of *Rhizoctonia solani*

Metabolites of the *R. solani* mycelia treated with respiratory inhibitors were subjected to GC-MS analysis. Around 190 to 260 metabolite peaks in each group of samples were detected after deconvolution, including sugars, amino acids, organic acids, alcohols, alkanes, and organic esters. Total ion chromatograms (TIC, Supplementary Figure 1) showed a significant difference of metabolic fingerprinting between control group and the fungicide treatment with RSD of the internal standard substance salicin of 3.2%, indicating an acceptable stability of the method. The RSDs of metabolites in the six technical replicates ranged from 3.4% to 180%.

### Hierarchical Cluster Analysis of Energy Synthesis Inhibitors Based on Metabolic Fingerprinting of *Rhizoctonia solani*

**FIGURE 1:**
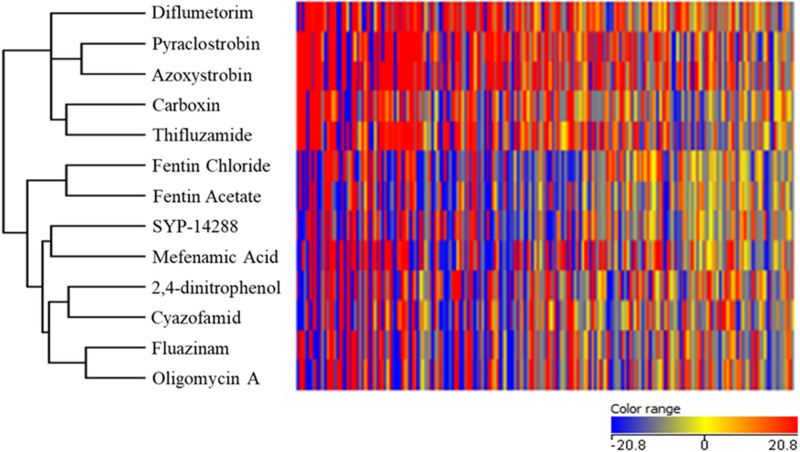
Hierarchical cluster analysis based on metabolic fingerprinting of *Rhizoctonia solani* treated with fungicides belonging to different types of mode of action.

Different metabolites with significant changes in *R. solani* were observed when treated with respiratory inhibitor, and an HCA of respiratory inhibitors was performed according to these compounds (Figure 1). Two distinct groups were generated by the HCA. The oxidation inhibitors including diflumetorim, pyraclostrobin, azoxystrobin, carboxin, and thifluzamide were clustered into one branch, which were further separated into three subgroups. The first subgroup contained diflumetorim with inhibition of complex I, the second subgroup contained carboxin and thifluzamide with inhibition of complex II, and the third subgroup contained pyraclostrobin and azoxystrobin with inhibition of complex III.

The phosphorylation inhibitors fentin chloride, fentin acetate, SYP-14288, mefenamic acid, 2,4-dinitrophen, cyazofamid, fluazinam, and oligomycin A were clustered into the second branch, which contained two subgroups. Fentin chloride and fentin acetate with inhibition on ATP synthase belonged to the first subgroup. However, SYP-14288, mefenamic acid, 2,4-dinitrophen, cyazofamid, fluazinam, and oligomycin A belonged to the second subgroup. In this subgroup, among the known MoA fungicides, only oligomycin A has inhibition on ATP synthase and cyazofamid with inhibition of complex III, while others are all uncouplers. From being clustered to phosphorylation process group by metabolic fingerprinting, SYP-14288 was speculated to have uncoupling activity or inhibition on ATP synthase.

Based on *t*-test and qualitative analysis on metabolites, biomarkers were selected according to the results showing the same regulation pattern under the treatment of either oxidation or phosphorylation inhibitors. Metabolites L-alanine, β-alanine, L-glutamine, cystamine, and 2-cortolone were identified as biomarkers for oxidative inhibitors, which were all up-regulated under oxidation inhibitors treatments (Table 2). The metabolite galactinol was up-regulated, and D-(+)-trehalose was down-regulated. Both of them were selected as biomarkers for phosphorylation inhibition (Table 3).

**TABLE 2:** Selected biomarkers for oxidation inhibitors.

Metabolite biomarker^*a*^	M/Z^*b*^	RT (min)^*c*^	Score^*d*^	Log FC ^*e*^ of fungicide				
				Diflumetorim	Pyraclostrobin	Azoxystrobin	Carboxin	Thifluzamide
L-Alanine	116.09	7.19	89.35	1.33	1.08	1.20	1.96	2.11
β-Alanine	248.13	11.74	90.93	3.93	1.80	3.77	6.93	6.62
L-Glutamine	156.09	15.82	83.91	2.60	2.39	2.42	3.97	4.47
Cystamine	218.11	20.11	84.70	15.38	11.92	11.19	15.84	13.14
2-Cortolone	73.04	20.69	86.78	18.06	16.07	15.86	17.81	16.82

*^*a*^Metabolite having the same regulation pattern.^*b*^M/Z, Mass-to-charge ratio.^*c*^RT, Retention time.^*d*^Score: Matching degree of metabolites with compounds from Fiehn library.^*e*^FC: change of metabolite contents between oxidation inhibitors treatment and non-treated control, which was confirmed by *t*-test.*

**TABLE 3:** Selected biomarkers for phosphorylation inhibitors.

Metabolite biomarker^*a*^	M/Z^*b*^	RT (min)^*c*^	Score^*d*^	Log FC^*e*^					
				SYP-14288	Fluazinam	2,4-dinitrophen	Mefenamic acid	Cyazofamid	Oligomycin A
Galactinol	204.10	25.93	84.99	1.80	20.50	24.60	1.90	1.10	20.67
D-(+) trehalose	361.18	24.77	89.38	–25.99	–24.94	–25.36	–1.14	–25.04	–25.19

*^*a*^Metabolite having the same regulation pattern.^*b*^M/Z, Mass-to-charge ratio.^*c*^RT, Retention time.^*d*^Score: Matching degree of metabolites with compounds from Fiehn library.^*e*^FC: change of metabolite contents between oxidation inhibitors treatment and non-treated control, which was confirmed by *t*-test.*

### Effect of SYP-14288 on ATP Production in *Rhizoctonia solani*

**FIGURE 2:**
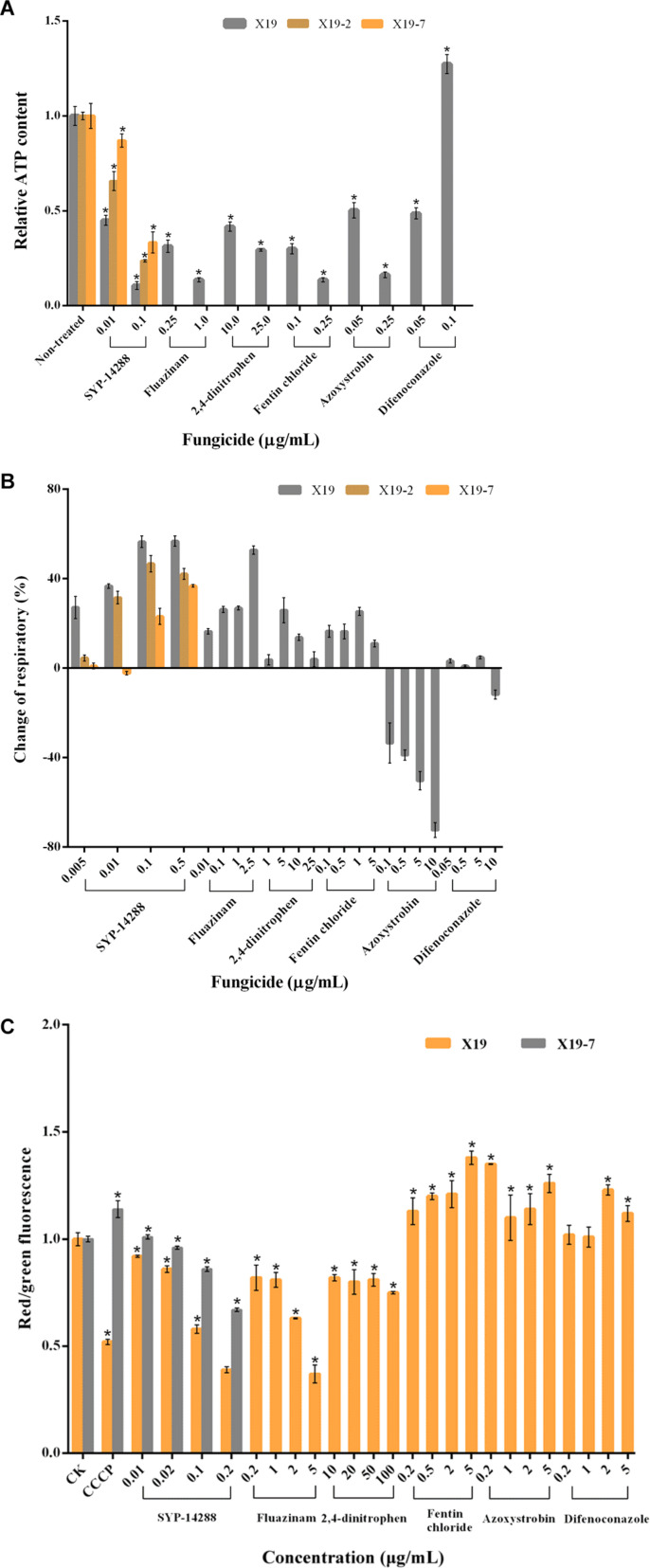
Respiration and growth of *Rhizoctonia solani* treated with fungicides belonging to different types of mode of action. **(A)** Relative ATP content of mycelia of *R. solani* wild-type isolate X19 treated with dimethyl sulfoxide (DMSO) and select fungicides. **(B)** Changes in respiratory rate of X19 mycelia treated with SYP-14288, fluazinam, 2,4-dinitrophen, fentin chloride, azoxystrobin, and difenoconazole. **(C)** Mitochondrial membrane potential (MMP) of *R. solani* X19 treated with various concentrations of fungicides. For ATP content and respiratory rate, SYP-14288-resistant mutants X19-2 and X19-7 were treated by SYP-14288 only. For MMP, SYP-14288-resistant mutant X19-7 was treated by SYP-14288 only. The change of MMP was expressed as a ratio of red and green fluorescence intensity. *R. solani* mitochondria were treated with dimethyl sulfoxide (DMSO; CK) or carbonyl cyanide *m*-chlorophenylhydrazone (CCCP) at 20 μmol/L. Values are the mean of three replicates ± standard error. The asterisk (*) indicates a significant difference based on one-way ANOVA (*P* < 0.01).

ATP contents were significantly reduced when the mycelium of *R. solani* X19 was treated with the uncouplers fluazinam and 2,4-dinitrophen, ATP synthase inhibitor fentin chloride, and complex III inhibitor azoxystrobin (Figure 2A). The reduction was positively correlated to fungicide concentration. Likewise, SYP-14288 had a similar trend in reducing ATP contents in wild-type isolate X19. For the two resistant mutants X19-2 and X19-7, ATP content was also reduced when treated with SYP-14288. However, the change of ATP content in the mycelium of X19-7 was smaller than X19-2 under the same treatment concentration of SYP-14288. When treated with difenoconazole at a concentration of 0.05 μg/ml, ATP content was sharply reduced in wild-type isolate X19. However, difenoconazole significantly promoted ATP synthesis of X19 at 0.1 μg/ml.

### Effect of SYP-14288 on *Rhizoctonia solani* Respiratory

Oxygen consumption of wild-type isolate X19 was measured under treatments with fungicides at different concentrations (Figure 2B). Fluazinam significantly increased the respiratory of *R. solani* X19, and the most change of respiratory rate reached 50%. 2,4-dinitrophen and fentin chloride effectively promoted respiration of *R. solani* in X19 at some concentrations. However, at a higher concentration, the respiratory rate decreased. Respiration was significantly inhibited by the respiratory inhibitor azoxystrobin, and the inhibitory effect was enhanced as the concentration increased. Respiration was not affected by the demethylation inhibitor difenoconazole. SYP-14288 significantly increased the rate of respiratory in *R. solani*, which was consistent with the uncouplers fluazinam and 2,4-dinitrophen and ATP synthase inhibitor fentin chloride. However, the change of respiratory rate was in a relatively stable trend along with the concentration increase compared with the above fungicides. Under most concentrations, respiratory rates of SYP-14288-resistant mutants X19-2 and X19-7 were increased by SYP-14288, but the change was lower than their parental strain X19.

### Effect of SYP-14288 on Mitochondrial Membrane Potential of *Rhizoctonia solani*

Fluazinam and 2,4-dinitrophen reduced MMP of *R. solani*, and the inhibition was positively correlated with fungicide concentration. At the highest concentration, the inhibition was significantly lower than that of DMSO and CCCP treatments (Figure 2C). Fentin chloride and azoxystrobin improved MMP of *R. solani*, which increased as the concentration increased. Difenoconazole had no effects on MMP of *R. solani* at low concentrations (0.2 and 1 μg/ml); however, MMP was improved at concentration of difenoconazole higher than 0.2 and 1 μg/ml. SYP-14288 significantly reduced MMP of wild-type isolate X19, and this effect of inhibition increased as fungicide concentration increased, which was consistent with uncouplers fluazinam and 2,4-dinitrophen. In comparing with strain X19, SYP-14288-resistant mutant X19-7 MMP increased significantly when treated with SYP-14288 and CCCP under the concentration of 0.02, 0.1, and 0.2 μg/ml.

## Discussion

We have demonstrated that metabolic fingerprinting had a high capacity in grouping fungicides by analyzing the production of inhibiting proteins involved in respiration. By using this method, we were able to place 10 fungicides into two clusters. These two main groups were highly correlated with the MoA defined by FRAC (Brent and Hollomon, 2007). The model successfully discriminated respiration inhibitors into subgroups of oxidation inhibitors (and also distinguished complex I, II, and III inhibitors) and phosphorylation inhibitors (ATP synthase inhibitors and uncouplers), demonstrating that the inhibition of oxidation and phosphorylation had a significant difference of metabolic profile of *R. solani* in mitochondrial respiration.

We identified the MoA of SYP-14288 by comparing the differences of metabolic fingerprinting of *R. solani* with HCA. SYP-14288 was closely related to mefenamic acid. It might act as an uncoupling agent, and possibly an ATP synthase inhibitor, as it significantly reduced ATP content and MMP of *R. solani* and increased respiratory rates. This activity matched the role of a canonical uncoupler.

The approach of metabolomics has some limitations and pitfalls in identifying MoAs (Aliferis and Chrysayi-Tokousbalides, 2011). It may not have a high resolution to distinguish MoAs of fungicides, which share a similar output on metabolic profiles of pathogen, such as uncouplers and ATP synthase inhibitors. In the case of cyazofamid on oomycetes, cyazofamid blocks electron transfer in the mitochondrial cytochrome bc1 complex by binding the Qi center of the enzyme (Mitani et al., 2001). However, based on the limited reports, it is not clear if there is a different MoA involved in cyazofamid. In distinguishing MoAs of fungicides, instead of directly analyzing all metabolite information, an improved and reliable way is to establish a model based on the profile of metabolites corresponding to specific genetic changes (Hu et al., 2019). In the meantime, metabolomics combined with other “omics” methods can greatly enhance the resolution of metabolomic models.

In order to determine the effect of uncouplers, ATP synthase inhibitors, and complex inhibitors on the respiration of *R*. *solani*, ATP content, respiration rate, and MMP were measured. Oxidative phosphorylation is defined as a process of electronic transference through an intricate assembly of more than 20 discrete carriers that is coupled to the synthesis of ATP (Lavin et al., 2008). As the primary energy-producing pathway in aerobic organisms, oxidative phosphorylation takes place in the inner membrane of mitochondria and is the most important process for energy production and survival (Marcethouben et al., 2009). Uncouplers of oxidative phosphorylation in mitochondria are capable of breaking the coupling between the electron transport chain and phosphorylation reactions and thus inhibiting ATP synthesis. As a result, energy is lost, but respiration rate and oxygen consumption increase (Vitoratos, 2014; Childress et al., 2020). In addition, the MMP decreases due to the proton potential through the inner membrane of mitochondria that is disturbed by uncouplers, which inhibit the reflux of protons through ATP synthase without affecting electron transport (Ding et al., 2019). We showed that SYP-14288 effectively inhibited ATP synthesis, promoted respiratory rate, and reduced MMP of both wild-type isolate and resistant mutants of *R. solani*, implying that SYP-14288 had uncoupling activity.

In conclusion, metabolic fingerprinting is an effective tool for quick identification of MoAs. The MoA of SYP-14288 on *R. solani* is possibly involved in affecting the uncoupling oxidative of phosphorylation.

## Data Availability Statement

The raw data supporting the conclusions of this article will be made available by the authors, without undue reservation.

## Author Contributions

PL, XL, JH, and LL designed the experiments. LL and XL performed the experiments. JL and BL participated in the work of toxicity determination. XC, TD, and ZW participated in data analysis. LL and XC wrote the manuscript. All authors reviewed the manuscript.

## Conflict of Interest

The authors declare that the research was conducted in the absence of any commercial or financial relationships that could be construed as a potential conflict of interest.

## Supplementary Material

Supplementary Figure 1 Total ion chromatograms (TIC) of *Rhizoctonia solani* X19 metabolome treated with DMSO **(A)** or SYP-14288 **(B)**.
