# Supplementary figures and images for: Metabolic Fingerprinting for Identifying the Mode of Action of the Fungicide SYP-14288 on Rhizoctonia solani
Source: Front Microbiol. 2020 Dec 9;11:574039. doi: 10.3389/fmicb.2020.574039 (PMC7755717; doi:10.3389/fmicb.2020.574039)

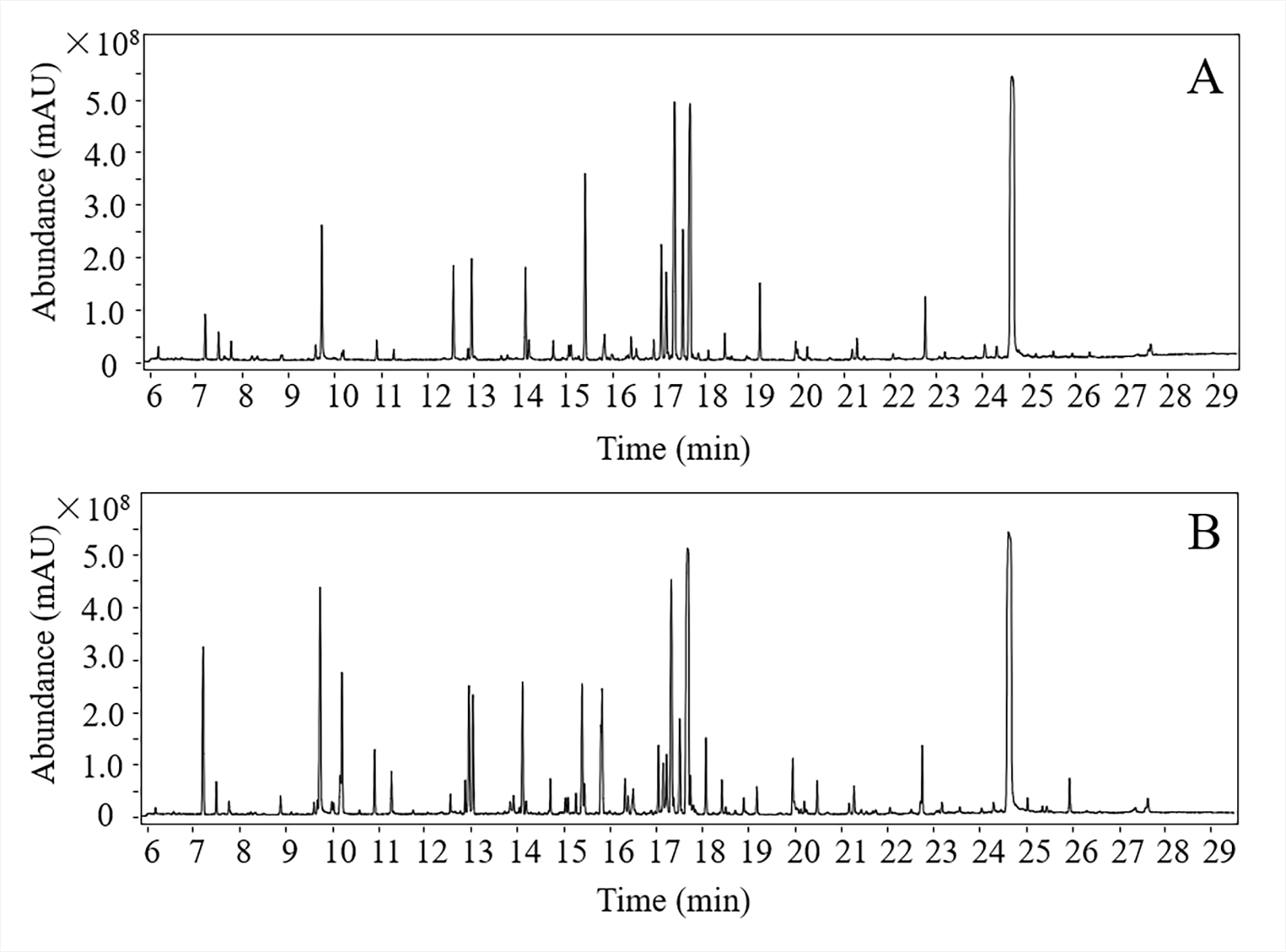

Supplement: Supplementary Figure 1 — Total ion chromatograms (TIC) of Rhizoctonia solani X19 metabolome treated with DMSO (A) or SYP-14288 (B). [file Image_1.TIF]
